# The study of Positive and Negative Affect in children and adolescents: New advances in a Spanish version of the PANAS

**DOI:** 10.1371/journal.pone.0221696

**Published:** 2019-08-27

**Authors:** Javier Ortuño-Sierra, Marta Bañuelos, Alicia Pérez de Albéniz, Beatriz Lucas Molina, Eduardo Fonseca-Pedrero

**Affiliations:** 1 Department of Educational Sciences, University of La Rioja, Logroño, Spain; 2 Department of Psychology, University of Valencia, Valencia, Spain; University of Lleida, SPAIN

## Abstract

The study of affective disorders among young population has become increasingly relevant in the last years. The PANAS is a widely used questionnaire devoted to assess positive and negative affect. The main purpose of the current study was to examine the psychometric properties of the Spanish version of the PANAS for children (PANAS). The sample consisted of 1032 children and adolescents aged between 10 and 15 years. The ESEM two factor model (Positive Affect and Negative Affect) was found as the most suitable model. The PANAS scores also showed acceptable internal consistency. The ESEM two factor model was invariant across gender and educational level. Results showed statistically significant differences in the latent mean scores with females scoring higher than males in and younger students scoring lower in PA. Positive and negative affect were related with external variables of well-being. The present psychometric study supports the PANAS as a brief and useful tool for the screening of PA and NA in children and adolescents.

## Introduction

The identification of emotional and behavioural problems during adolescence and before to clinical transition, is receiving more and more attention nowadays [1]. In particular, recent studies have shown that anxiety and depression are at this moment one of the leading causes of disability in European countries [2,3]. Furthermore, negative outcomes have been associated with anxiety and depression, not only in adult population but in adolescent and child populations as well [3–5]. For instance, it has been documented that anxious children and adolescents experiencing poor peer relations are described as withdrawn and shy by their classmates [6].

Among the different instruments devoted to assess emotional and affect impairments, the Positive and Negative Affect Schedule (PANAS) [7] is one of the most extensively-used instruments. One of the strong points of the PANAS is that it allows measuring Positive Affect (PA) and Negative Affect (NA) [7,8] being, at the same time, easy to administer. Positive affect is related to the experiencing of a positive mood, with feelings such as joy, interest, enthusiasm, and alertness [7]. Negative affect reflects emotional distress, and includes moods like fear, sadness, anger and guilt [7]. As proposed by Watson and Tellegen [9], NA may be the general component of anxiety and depression and lower levels of PA is specifically characteristic of depression. Later, the introduction of a new factor, the physiological hyperarousal (PH), explained anxiety as a combination of higher levels of both NA and PH [8]. Positive and Negative affect have been related to relevant psychological constructs like social functioning, affect, and personal well-being [10–12].

Watson, Clark, and Tellegen [7] indicated that the PANAS is a pure measure of PA and NA considered as independent constructs. However, some other studies have found negative associations leading to affirm that PA and NA are negatively associated constructs [13–16]. Initially, and due to the weak and negative correlation, Watson et al. [7] stated that the PANAS had an orthogonal two factor structure. On the other hand, some other studies supported an oblique model with PA and NA as separate but also moderately associated constructs [13,17]. For instance, Crawford and Henry [13], showed that the best model fit was reached after PA and NA were allowed to correlate, what from a psychometric point of view seems predictable, as more complex models are prone to reveal better fit indices.

Also, and despite the evidence about the two-factor structure [18–20], some others studies reveal a three-factor or a higher order model structure [15,21–23]. With this regard, Mehrabian [23], suggested the division of the NA dimension into two separate factors: Afraid (scared, nervous, afraid, guilty, ashamed, and jittery) and Upset (distressed, irritated, hostile, and upset), with PA as an independent factor. It has also been suggested that both the two and the three-factor models provide good fit, with the three-factor structure showing better fit [15,22,24]. For instance, Ortuño-Sierra et al. [15] with a large sample of Spanish adolescents, revealed that the three-factor structure with some modifications, and allowing PA and NA to correlate, was the structure that better fit the data. However, other studies in Spain indicated that the two factor model was the best fitting model with the three-factor model showing a poor adjusting in adolescents, adult, and elderly population [18]. Thus, studies are not conclusive about the manifestation of positive and negative affect, and new research analyzing the factor structure of the PANAS is still needed. Recently, new contributions have added relevant about the Spanish version of the PANAS [25,26]. A short version of the PANAS for children was validated and a two factor structure was found as the most satisfactory solution [25]. In addition, a bifactor model has been proposed as the best model explaining the PANAS structure [27]. The bifactor model incorporates the traditional PA and NA as uncorrelated factors as well as an additional general factor.

Considering that the previous research is still not conclusive, the inclusion of relatively new techniques such as the Exploratory Structural Equation Modelling (ESEM) [28] could add relevant information in order to better comprehend the underlying dimensional structure of the PANAS. The ESEM approach, contrary to the traditional Structural Equation Models (SEM), permits to analyze measurement models without the restrictions of the CFA models, like, for instance, constraining all cross loading to zero. Thus, factor loadings of all factors are calculated for each item. This, may better represent the latent construct of affect in terms of a psychological point of view. With this regard, recent studies have analyzed the structure of positive and negative affect by means of the ESEM approach [29,30]. Therefore, and as proposed by Ortuño-Sierra et al. [31], studies analyzing the factor structure of the PANAS are still needed.

Moreover the study of relevant aspect of the factor structure of the PANAS, as it is the study of Measurement Invariant (MI) across groups remains still unclear. If MI is not sustained, interpretation of the data could be erroneous [32]. However, there is a lack of studies examining if the dimensional structure of the PANAS is invariant across relevant variables like gender, age or educational level [15,33,34]. In addition, the relation of the PANAS with external variables has been analyzed [35,36]. For instance, Serafini et al. [36] found a strong correlation among the PANAS and substance abuse.

It is worth noting, that the tripartite model has been also tested in children and adolescents [15,35,37–40]. For instance, in the study of Lonigan et al. [40] lower levels of PA discriminated those children suffering for depression and those with an anxiety disorder. On the other hand, children with higher level of anxiety and depressive symptoms revealed similar levels of NA. With this regard, there is a children version of the PANAS, the PANAS-C [41] that allows measuring PA and NA in children and adolescent. The PANAS-C was developed based on the original PANAS but is composed of 27 items, 15 belonging to the NA subscale and 12 to the PA subscale. The reliability of the scores and different evidences of validity have been reported in previous studies [33,39,41,42]. The PANAS-C has been translated into Spanish and its psychometric properties have been studied [43] in South America (Peru), although the different Spanish dialects does not preclude multiple Spanish-language scales for assessing PA and NA among youth. A Spanish version of the PANAS for children, the PANAS-N (Positive and Negative Affect Schedule-Niños) [35], revealed a two-factor structure with a negative and low correlation between PA and NA. Reliability of the scores was appropriate and some other sources of validity evidences like the relation with external variables were adequate. Nonetheless, the three options Likert-type response scale could be affecting the reliability of the instrument.Nonetheless, the original PANAS has shown its adequacy for its use in children and adolescent populations, revealing a three factor structure as the most satisfactory [44].

In addition, considering the widespread use of the PANAS across the world, the relevance of screening aspects like positive and negative affect in children and adolescence populations, and the lack of studies about the adequacy and internal structure of the PANAS in these populations in Spain, more validity evidences about its structure are still required in order to its use in children and adolescents. Studying and stablishing evidences of validity and reliability of dimensional measures is relevant and closely related to the Research Domain Criteria (RDoC) [45]. This study, thus, aimed to serve the RDoC and similar empirical initiatives.

Within this research framework, the main objective of the present work was to analyze the psychometric properties of the PANAS in a representative sample of nonclinical children and adolescents. In particular, we sought to investigate: a) the internal structure of the PANAS by means of SEM and ESEM models; b) the internal consistency of the PANAS scores through McDonald’s Omega; c) to test the MI across gender and educational level; and d) to study evidences of validity of the instrument with personal well-being. Consistent with previous studies, we hypothesized that a two or a three-factor solution would provide the best fit with the data. We also hypothesized that PA and NA would have adequate internal consistency. Previous studies conducted with the PANAS indicated that Positive and Negative affect dimensions are invariant across gender, age, and educational level [15,33], therefore, we also hypothesized that the factor structure underlying the PANAS would be invariant across gender and educational level and that the PANAS would be related to personal well-being.

## Method

### Participants

A total of 1032 non-clinical adolescents volunteered to participate in the study (convenient samples), 531 were male (51.5%). Participants ages ranged from 10 to 15 (*M* = 11.91; *SD* = 1.37). Concerning age, the sample distribution was as follows: 10 years old (*n* = 184; 17.8%), 11 years old (*n* = 271; 26.3%), 12 years old (*n* = 210; 20.3%), 13 years old (*n* = 224; 21.7%), 14 years old (*n* = 112; 10.9%, 15 years old (*n* = 31; 3%). A total of 527 students (51%) belonged to the Secondary level (ESO) and 505 (49%) belonged to a lower educational level (Primary Education) attending to the Spanish educational system.

### Instruments

Positive and Negative Affect Schedule for children (PANAS) [7]. The PANAS, is a self-reported adjective checklist that contains two 10-item subscales designed to measure positive (i.e., active, alert, attentive, determined, enthusiastic, excited, inspired, interested, proud, and strong) and negative affect (i.e., afraid, ashamed, distressed, guilty, hostile, irritated, jittery, nervous, scared, and upset). The PANAS uses a Likert-type scale, with answers ranging from 1, *very slightly or not at all*, to 5, *extremely or very much*. The Spanish version of the PANAS [46], translated and adapted according to the international guidelines [47] and with a 5 Likert-type scale was used. The Spanish version of the PANAS has shown evidences of internal consistency with scores ranging from .86 to .90 for PA, and from .84 to .87 for NA [15]. Correlations between the two scales ranged from -.12 to -.23 [15].

The Personal Wellbeing Index- School Children (PWI-SC) [48]. The PWI-SC is derived from the Personal Wellbeing Index for Adult population (PWI-A). The PWI-SC contains eight items of satisfaction, corresponding to different quality of life domain: standard of living, health, life achievement, personal relationships, personal safety, community-connectedness, future security and spirituality-religion. The PWI-SC was modified in order to make the wording more accessible for children and adolescents. In addition, satisfaction was substitute by happiness. Psychometric properties of the instrument have been confirmed in previous studies [49]. In order to its use in Spanish population, the translation and adaptation of the instrument were carried out using the back-translation procedure, following the international guidelines of the International test Commission [50,51]. The PWI-SC showed adequate levels of internal consistency of the scores, with Ordinal alpha values of .80.

### Procedure

Participants assent to take part in the study. For those participants under the age of 18, informed consent was obtained from parents. The volunteers filled in the questionnaires in a neutral and relaxed state without any mood induction. The study was carried out in groups of 10 to 30 students during normal lecture hours. The study was presented to the participants as a research project on diverse personality traits. Participants were informed about the voluntary nature of their participation and they were given assurances of the confidentiality of their responses. They received no type of incentive for taking part. Administration of the measurement instruments was always under the supervision of a researcher.

### Ethics statement

Parents or legal tutors were asked to provide written informed consent in order for their children to participate in the study. Participants were informed of the confidentiality of their responses and of the voluntary nature of the study. No incentive was provided for their participation. Administration took place under the supervision of researchers. The study was approved by the research and ethics committee at the University of La Rioja. The investigation must have been conducted according to the principles expressed in the Declaration of Helsinki.

### Data analyses

Different analysis were carried out in the study. With the intention to analyze the internal structure of the PANAS, we conducted several CFAs to at the item level. The weighted least squares means and variance adjusted (WLSMV) estimator and the polychoric correlation matrix were used, attending to the categorical nature of the variables. We used the oblique Geomin rotation with epsilon value = 0.5 [28,52]. The chi-square (χ^2^), the Comparative Fit Index (CFI), the Tucker-Lewis Index (TLI), and the root mean square error of approximation (RMSEA), and its 90% confidence interval and the Weighted Root Mean Square Residual (WRMR) were considered as goodness-of-fit indices. In order to achieve a good fit of the data to the model, the values of CFI and TLI over 0.95 are considered adequate and over .90 acceptable, and the RMSEA values should be under 0.08 for a reasonable fit and under 0.05 for a good fit [53–55]. For the WRMR, lower values than 1.0 have been reported as adequate [55].

First, we tested the one-factor model. In order to test whether PA and NA behave as two distinctive factors, a two-factor model was tested, and also a two-factor model with PA and NA forced to be orthogonal, following Watson and Clark [7] first hypothesis. According to hierarchical model suggested by Mehrabian [23], NA was conceptualized as a second order-factor with two distinct factors, upset and afraid. Thus, we studied a three-factor solution, and a derived three factor solution with PA and NA as uncorrelated. The last model to be tested was a bifactor solution, suggested to be more satisfactory in previous studies [15,24,56]. Finally, two ESEM models with a two and a three factor structure were also tested. The ESEM models allow all loadings to be freely estimate. Worth noting, and considering previous studies including correlated errors [15,24], items from the same content categories were allow to correlate in all the models explained above.

Next, we calculated descriptive statistics for the dimensions of the PANAS. In addition, McDonald's Omega was calculated as a measured of the internal consistency of the scores.

Next, in order to test measurement invariance (MI), successive multigroup CFAs were conducted [32]. Attending to the limitations of the Δχ^2^ regarding its sensitivity to sample size, we followed the change in CFI (ΔCFI), proposed by Cheung and Rensvold [57] as a more suitable criterion, to determine whether nested models are practically equivalent. Latent mean differences across gender and educational level groups were estimated, fixing the latent mean values to zero in the male and in the Primary students groups. For comparisons among groups in the latent means, statistical significance was based on the *z* statistic. The group in which the latent mean was fixed to zero was considered as the reference group [58].

Finally, and with the aim of studying if positive and negative affect were related to personal well-being, correlation between the PANAS and the PWI-SC scores was calculated by means of Pearson correlation.

SPSS 22.0 [59], Mplus 7.4 [60], and FACTOR 8.0 [61] were used for data analysis.

## Results

### Evidences of internal structure validity

As can be seen in Table 1, the ESEM three factor model was the only one over the .95 CFI criterion. In addition, the bifactor model and the ESEM two factor model revealed a CFI above .90. The ESEM three factor model revealed a structure with the PA dimension and the NA dimension separated into two separate dimensions with one dimension composed of two items. In addition, cross loading (see Table 2) were found between the two NA dimensions displayed (NA 1 and NA 2). The ESEM two factor model was related to the PA and NA dimensions. Substantial correlations were found between items 9 (*enthusiastic*), 3 (*inspired*) and 1 (*interested*) in all the models studied. Hence, correlations between items 1 and 3, item 1 and 9, and item 3 and 9 were allowed. Some other correlated errors were identified. However, we decided to compute just 3 out of 180 potential correlated errors based on parsimony criterion and considering those items with the same psychological content. Different improvements were found in all the models. In particular, the three-factor model oblique solution, obtained adequate goodness-of-fit indices when correlated errors were included. In addition, fit indices for ESEM and bifactor models also were higher when correlated errors were considered. However, and due to the critics raised with regards to the use of this approach [62], we decided to choose between those models that showed an adequate fit without the inclusion of correlated errors.

**Table 1:** Goodness-of-fit indices of the dimensional models tested.

Models	χ^2^	*df*	CFI	TLI	RMSEA (90% C.I.)	WRMR
One-dimensional model	2740.57	170	.52	.46	.12 (.12-.13)	3.30
One-dimensional model with correlated term	2318.55	162	.68	.62	.11 (.11-.12)	3.14
Two-factor model orthogonal (Watson and Clarck)	874.41	170	.87	.85	.06 (.06-.07)	1.99
Two-factor model orthogonal, CE permitted (3)	789.80	167	.88	.87	.06 (.06-.07)	1.89
Two-factor model oblique solution	812.08	169	.88	.87	.06 (.06-.07)	1.73
Two-factor model oblique solution, CE permitted (3)	684.43	166	.89	.89	.05 (.05-.06)	1.57
Three-factor model oblique solution	743.32	167	.89	.88	.06 (.05-.06)	1.64
Three-factor model oblique solution, CE permitted (3)	616.52	164	.92	.90	.05 (.05-.06)	1.48
Three-factor model PA and NA uncorrelated	844.74	169	.87	.86	.06 (.06-.07)	1.69
Three factor model PA and NA uncorrelated, CE permitted (3)	760.20	166	.89	.87	.06 (.05-.07)	1.86
Bifactor model	566.99	150	.92	.90	.05 (.05-.06)	1.33
Bifactor model, CE permitted (3)	479.69	147	.94	.92	.05 (.04-.05)	1.21
ESEM two factor solution	666.01	151	.90	.88	.06 (.05-.06)	1.33
ESEM two factor solution, CE permitted (3)	561.31	148	.92	.90	.05(.05-.06)	1.22
ESEM three factor solution	411.06	133	.95	.93	.04 (.04-.05)	0.99
ESEM three factor solution, CE permitted (3)	358.31	130	.96	.94	.04(.04-.05)	0.92

*Note*. χ^2^ = Chi square; *df* = degrees of freedom; CFI = Comparative Fit Index; TLI = Tucker-Lewis Index; RMSEA = Root Mean Square Error of Approximation; CI = Confidence Interval; WRMR = Weighted Root Mean Square Residual.

**Table 2:** Standardized factor loadings for the ESEM three and two factor models.

Items	Loadings		Items	Loadings	
ESEM three factor model	PA	NA	ESEM two factor model	PA	NA
**PA**			**PA**		
1. Interest	.30	-.02	1. Interest	.35	.13
3. Excited	.52	-.01	3. Excited	.58	.20
5. Strong	.55	-.18	5. Strong	.58	-.09
9. Enthusiastic	.51	.03	9. Enthusiastic	.57	.22
10. Proud	.58	-.08	10. Proud	.61	-.01
12. Alert	.54	-.03	12. Alert	.52	-.04
14. Inspired	.51	.11	14. Inspired	.51	.15
16. Determined	.61	.03	16. Determined	.57	-.01
17. Attentive	.54	-.02	17. Attentive	.55	.01
19. Active	.56	-.09	19. Active	.57	-.05
**NA 1**		.	**NA**		
7. Scared (NA 2)	.48 (.34)	.08	2.Distressed	.54	.01
13. Ashamed (NA 2)	.35 (.24)	-.03	4. Upset	.59	-.12
**NA 2**			6. Guilty	.49	-.03
2. Distressed	.56	.03	7. Scared	.50	.04
4. Upset	.71	-.01	8. Hostile	.60	-.13
6. Guilty	.52	.03	11. Irritable	.45	-.15
8. Hostile	.67	-.04	13. Ashamed	.38	.01
11. Irritable	.46	-.11	15. Nervous	.56	.04
15. Nervous	.51	.06	18. Jittery	.54	.07
18. Jittery	.52	.12	20. Afraid	.55	-.01
20. Afraid (NA 1)	.45 (.32)	-.02			
Factor Correlations					
PA with NA1 = .01; PA with NA 2 = -.20; NA 1 with NA 2 = .15			PA with NA = -.22		

*Note*. All standardized factor loadings estimated were statistically significant (*p* < .01). (NA 2 and NA 1) = significant cross loadings of NA 2 and NA 1 in the other NA dimension.

We, then, studied the standardized factor loadings in these three models. In this case, the bifactor model showed nine non-significant factor loadings, and, in addition, standardized factor loadings were lower than in the ESEM two and three factor models. For this reason, we decided not to consider the bifactor model as adequate. The study of the factor loadings in the ESEM models revealed significant cross loadings of items 7 (*scared*) and 20 (*afraid*) when the three factor was considered. No cross loading were found for the ESEM two factor solution. Attending to the CFI over .90, the RMSEA values under .08, and the WRMR close to 1.0 found in the ESEM two factor model, the cross loadings found in the three factor model, the fact that a dimension with two items is not recommended, and the lack of psychological interpretation of the three factor model, we decided to choose the ESEM two factor model over the ESEM three factor model. Thus, we studied MI and the internal consistency of the scores of this model. As shown in Table 3, all standardized factor loadings for the ESEM two and the three factor models were statistically significant with significant cross loading in the case of the three factor. Factor loadings, in the two factor model, for the PA factor ranged between 0.35 (*Interested*) and 0.61 (Proud) items. In the NA dimension, factor loadings ranged from 0.38 (*Ashamed*) to 0.60 *(Hostile)*. Correlations between factors was -0.22, being statistically significant (*p* < 0.01).

**Table 3:** Goodness-of-fit indices for measurement invariance of the PANAS (two factor ESEM model) across gender and educational level.

	χ^2^	*df*	CFI	TLI	RMSEA (90% C.I.)	WRMR	ΔCFI
**Gender**							
Male (*n* = 531)	407.75	151	.91	.88	.05 (.05-.06)	1.05	
Female (*n* = 501)	436.33	151	.89	.87	.06 (.05-.06)	1.13	
Configural Invariance	844.75	302	.90	.88	.06 (.05-.06)	1.54	
Strong factorial invariance	949.54	396	.90	.90	.05 (.05-.06)	1.84	-.01
**Educational Level (Primary Vs Secondary)**							
Primary 10–12 (*n* = 527)	453.64	151	.89	.86	.06 (.05-.06)	1.13	
Secondary 12–15 (*n* = 505)	410.44	151	.90	.86	.05 (.05-.06)	1.08	
Configural Invariance	863.69	302	.90	.87	.06 (.05-.06)	1.57	
Scalar invariance	868.21	396	.90	.91	.05 (-04-.05)	1.76	-.01

*Note*. χ^2^ = Chi square; *df* = degrees of freedom; CFI = Comparative Fit Index; TLI = Tucker-Lewis Index; RMSEA = Root Mean Square Error of Approximation; CI = Confidence Interval; WRMR = Weighted Root Mean Square Residual. ΔCFI = Change in Confirmatory Fit Index.

With regards to the orthogonality of PA and NA, the goodness-of-fit indices were similar in the oblique than in the orthogonal solution when the two-factor the three-factor SEM models were compared. In addition, the correlation between PA and NA in the ESEM two-factor model was negative and statistically significant.

### Study of the measurement invariance of the PANAS

Factorial equivalence of ESEM two factor model across a) gender (males versus females), and b) educational level (primary -10 to 12 years old- versus secondary students -13 to 15 years old) was studied. First, we tested whether the ESEM two factor model showed a reasonable good fit in each group. Next, configural and scalar invariance were examined (see Table 4). The comparison between the configural and scalar invariance models showed differences in CFI (ΔCFI) below .01 across gender and educational level. Thus, the hypothesis of MI was confirmed by these two variables

**Table 4:** Mean and standard deviation for the PANAS for the total sample, males and females, and Primary and Secondary students separately.

	Total (*N* = 1032)		Primary (*n* = 527)		Secondary (*n* = 505)		Male (*n* = 531)		Female (*n* = 501)	
	*M*	*SD*	*M*	*SD*	*M*	*SD*	*M*	*SD*	*M*	*SD*
NA	22.91	6.82	23.16	6.8	22.65	6.84	22.40	6.70	23.43	6.92
PA	38.46	6.13	37.26	6.31	39.72	6.13	37.82	6.41	39.14	5.75

### Comparisons in the latent means

The comparison across groups in latent means revealed statistically significant differences in the PANAS dimensions. Thus, the comparison across groups in latent means indicated that, on average, women scored 0.58 units over men in the NA dimension (*p* < 0.01). With regards to educational level, comparison across groups in latent means revealed statistically significant differences in PA. In particular, the older students, belonging to secondary education, scored 0.50 units over the younger students in PA (*p* < 0.01) and -0.16 units under the younger students in NA (*p* < 0.05). No other statistically significant differences were found neither for gender nor for educational level.

### Internal consistency and descriptive statistics

Taking into account that the ESEM two factor model was retained as the most suitable model considering the fit indices and the factor loadings, descriptive statistics and internal consistency for this model were calculated. The mean and standard deviation for the PANAS dimensions are shown in Table 1. Internal consistency values for the PANAS PA and PANAS NA scores were 0.84 and 0.88, respectively, estimated using McDonald's Omega.

### Correlation with external variables

Correlations between PANAS dimensions and PWI-SC total score were statistically significant. In particular, correlations between PA and NA and PWI-SC total score were .43 and -.29, respectively. As expected, personal well-being was positively associated with PA, and negatively associated with NA.

## Discussion

To date, there is a lack of studies analyzing the fruitfulness of the PANAS [7] for its use in children and adolescents at school in Spain. Thus, the main purpose of the study was to analyze the psychometric properties of the Spanish version of the PANAS [46] in children and adolescents. Therefore, we analyse the internal structure of the PANAS, estimated the reliability of the scores, and obtained different sources of validity evidence. The study of these kind of scales provides information in order to examine different psychological difficulties, both in children and adolescents, and are well-suited for the RDoC initiative of the NIMH [45]. Results revealed the adequate psychometric properties of the PANAS, allowing its use for screening PA and NA in young populations.

When compared, the two-factor model yielded better fit than the one-factor model suggesting that PA and NA are separate dimensions accordingly with previous research [7,9]. In addition, we studied the orthogonality of the PANAS. In this case, the correlation between PA and NA factors, derived from the ESEM two factor model, was negative and significant. Therefore, PA and NA should not be considered as independent factors, what is not in line with the author’s proposal [7].

Also, the three-factor model oblique solution was close to be appropriate but still poor. The three-factor model with correlated errors included revealed adequate goodness-of-fit indices. Similar results have been found in previous studies, suggesting that a better fit is reached after allowing this content categories to correlate [15,24]. In addition, a bifactor solution was tested [15,56]. It should be stressed that, although the bifactor model and the ESEM three-factor models displayed adequate goodness-of-fit indices, the ESEM two-factor model was preferred due to the lack of significance in nine factor loadings in the bifactor model and the fact that the ESEM three-factor model showed different cross loadings, revealed a dimension composed of only two items, and in addition there was a lack of psychological meaning. Also, the three-factor model oblique solution with correlated errors revealed adequate factor loadings and a meaningful structure. Nonetheless, we decide not to choose this model as some critics have been raised with regards to the use of correlated errors. For these reasons, the ESEM two-factor model was preferred. Similar to the results found in this study, it is worth mentioning that recent previous studies analysing the short version of the PANAS for children in Spain revealed the two factor as the most satisfactory [25]. It is worth mentioning, that new EFA approaches as the Hull method [63] allow for a better factor retention and may be considered for future studies with regards to the internal structure of the PANAS.

The study of the positive and negative affect by means of ESEM models is relatively new [29,30], and there is a lack research analyzing the PANAS structure with this approach, that allow analyzing the structure of positive and negative affect without the restrictions of the CFA models. The results, also revealed a ESEM three factor model as adequate, similar to previous research suggesting the adequacy of a three factor model [15,21,24], although in the present study, the dimensions found for the NA factor were composed of different items. In addition, different modification indices were found and the inclusion of correlated errors improved the goodness-of-fit indices of all the models studied. Therefore, caution should be applied, and more research is still needed in order to further analyze the PANAS structure and explain positive and negative affect manifestations in children and adolescents.

A bifactor solution, was also found as satisfactory, similar to the study of Ebesutani et al. [56], that found the bifactor model as the best solution, but still chose a two model solution in order to better understand the structure of the PANAS. As these authors posed, the fact that fear and distress behave as group factors may suggest that negative affectivity could be comprised largely of a fear and distress component among children and adolescents [56]. The PANAS intended to measure, originally, PA and NA. Nonetheless, the inclusion of a lower-order factor of NA allows for a more comprehensible interpretation of this factor that could help researchers and professionals of education, in order to analyse mood states and psychological aspects in children and adolescent students. Therefore, and as mention above, more studies analyzing the factor structure of the PANAS by means of ESEM models are needed in these populations.

In addition, McDonald’s Omega values were adequate when analyzing the internal consistency of the scores for ESEM two factor solution, being adequate for the NA dimension and good for the PA. Other studies have also shown adequate levels of internal consistency for the PANAS structure [15,25].

Another important issue, as it is the study of structural equivalence was also studied. The MI of the PANAS in the ESEM two factor model was tested across gender (male and female) and educational level (Primary and Secondary education). Structural equivalence was found for both, gender and educational level. These results are similar to other studies revealing total and partial factorial equivalence in the PANAS [15,24,33] across different groups. The study of Ebesutani et al. [56], for instance, revealed MI across gender, although differences between younger and older groups were found in terms of the bifactor model of NA avoiding the support of MI. The findings found in this study provide evidence about the possibility of stablishing proper comparisons across these two groups, that could be unfounded if it is not possible to guarantee that participants interpret and understand the latent construct in a similar manner [32].

In addition, when latent means were compared, females scored higher than males in NA. Concerning the educational level, students belonging to the superior level scored higher in PA than younger students. It is possible that as adolescents become older and transit to the next educational level, internalizing issues are less intense, considering the end of childhood as a critical period for affective reactions. Likewise, and with regard to gender, it is well known that males refer less emotional problems than females [64], what can explain the higher scores of females in NA.

Results from the analysis of the sources of validity evidence with external variables yielded a significant association between the PANAS dimensions and the PWI-SC scores. This is congruent with previous indicating that PA and NA are related with other variables like anxiety, depression, affect, and personal well-being [11,35,36]. The results found reveal the adequacy of PA and NA in order to be use as an indicator of, not only mental health, but also personal well-being in young populations. In this sense, screening of PA and NA could be relevant for the early detection in order to avoid psychological disorders that potentially could become permanent.

It is worth mentioning that this study present some limitations. First, measurement of PA and NA, as well as well-being was based solely on self-report and there are well-known inherent problematics like the effect of stigmatization, the possibility of misunderstanding of some items or the lack of introspection of some participants. Hence, future studies should consider the use of external informants, interviews or even bio-behavioral and/or biological markers. Second, no information was gathered regarding the participants´ psychiatric morbidity or the use or abuse of substances, aspects that may partially influence the results. Finally, the cross-sectional nature of this study prevent stablishing cause effect relations.

In conclusion, the PANAS revealed adequate evidences of validity. These results have clear implications for the research on the construct validity of the PANAS and for its use in school populations in order to study PA and NA in children and adolescents populations. In addition, the present study provides valuable information to better comprehend the structure of psychopathology across childhood and adolescence development, allowing the implementation of future treatments of negative emotions. Future research should continue to advance in the study of PA and NA in children and adolescent populations, as well as study the measurement invariance of the PA and NA dimensions across other variables such as race or culture.

## Supporting information

S1 File FINAL_baseMartaPANASYBIENESTARORTU_FINAL. (SAV)
